# Monoterpenol Oxidative Metabolism: Role in Plant Adaptation and Potential Applications

**DOI:** 10.3389/fpls.2016.00509

**Published:** 2016-04-26

**Authors:** Tina Ilc, Claire Parage, Benoît Boachon, Nicolas Navrot, Danièle Werck-Reichhart

**Affiliations:** Institut de Biologie Moléculaire des Plantes, Centre National de la Recherche Scientifique, Université de StrasbourgStrasbourg, France

**Keywords:** geraniol, linalool, cytochrome P450, oxidation, monoterpene indole alkaloids, iridoids, lilac aldehydes, aroma and fragrance

## Abstract

Plants use monoterpenols as precursors for the production of functionally and structurally diverse molecules, which are key players in interactions with other organisms such as pollinators, flower visitors, herbivores, fungal, or microbial pathogens. For humans, many of these monoterpenol derivatives are economically important because of their pharmaceutical, nutraceutical, flavor, or fragrance applications. The biosynthesis of these derivatives is to a large extent catalyzed by enzymes from the cytochrome P450 superfamily. Here we review the knowledge on monoterpenol oxidative metabolism in plants with special focus on recent elucidations of oxidation steps leading to diverse linalool and geraniol derivatives. We evaluate the common features between oxidation pathways of these two monoterpenols, such as involvement of the CYP76 family, and highlight the differences. Finally, we discuss the missing steps and other open questions in the biosynthesis of oxygenated monoterpenol derivatives.

## Introduction

**FIGURE 1:**
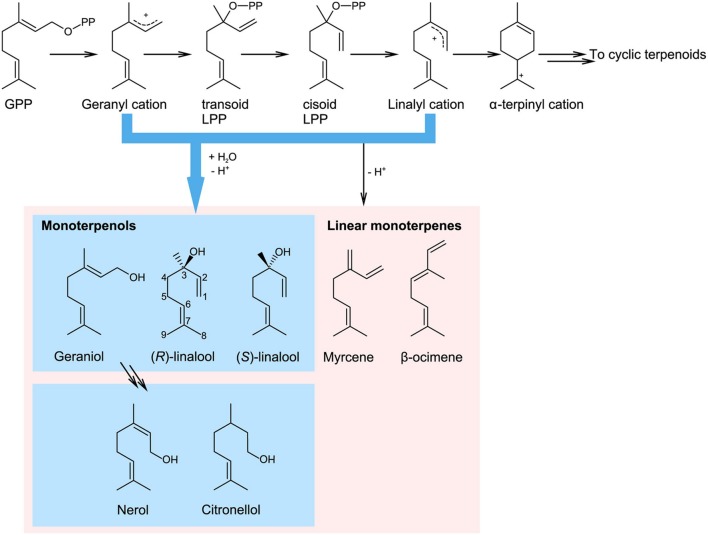
**Terpene synthase catalyzed biosynthesis of monoterpenols from geranyl diphosphate (GPP; adapted from Degenhardt et al., 2009).** Terpene synthases cleave the diphosphate group from the substrate, which results in the formation of a carbocation intermediate that can further rearrange. The reaction can be terminated by proton abstraction, which yields hydrocarbon monoterpenes (here illustrated by myrcene and β-ocimene), or by addition of water, which yields monoterpene alcohols, or monoterpenols. The route leading to monoterpenols is highlighted in green. (*E*)-8- and (*Z*)-8-linalool oxidized derivatives are also referred to as 8- and 9-linalool derivatives, respectively. In some older papers, (*E*)-8-hydroxygeraniol is referred to as 10-hydroxygeraniol. GPP, geranyl diphosphate; LPP, linalyl diphosphate.

Monoterpenols and their derivatives form a very diversified group of plant specialized metabolites with important ecological roles, such as flower or fruit scent. In addition, they act as precursors to numerous allelochemicals such as iridoids or monoterpenoid indole alkaloids (MIAs). They are also an economically important raw material in flavor or fragrance, nutraceutical and pharmaceutical industry. Monoterpenols belong to the large family of monoterpenes, which are compounds composed of two isoprenyl units (forming 10-carbon skeletons). Monoterpenes are products of terpene synthase enzymes, which convert a monoterpenyl diphosphate substrate (usually geranyl or neryl diphosphate, GPP or NPP, respectively) into a variety of structures. The reaction starts with ionization of monoterpenyl diphosphate, which yields a carbocation intermediate, prone to rearrangements and cyclisation (**Figure 1**). The carbocation then follows one of two fates, depending on the catalytic site of the terpene synthase. It either loses a proton and forms a cyclic or a linear hydrocarbon, or is attacked by hydroxyl ion (or a water molecule and looses a proton) to form a monoterpenol (Degenhardt et al., 2009). In addition, a terpene synthase-independent synthesis of geraniol was recently discovered in roses (*Rosa* ×*hybrida*; Magnard et al., 2015). This work revealed that a cytosolic enzyme Nudix hydrolase 1 (NUDX1) hydrolyses geranyl diphosphate and is required for the formation of geraniol in rose flowers.

The most common monoterpenols in plants are linalool, nerol, and geraniol, the latter two being *cis* and *trans* isomers, respectively (**Figure 1**). Linalool is an optically active compound, present in the flower scent of both monocotyledonous and dicotyledonous plants (Raguso and Pichersky, 1999). Both enantiomers, 3*S*- and 3*R*-linalool, occur naturally at enantiomeric excess ranging from 100% (3*S*) to 97.5% (3*R*) in different plant species (Aprotosoaie et al., 2014). Irregular monoterpenols, i.e., built from non-head-to-tail isoprenyl condensation, such as chrysanthemol, lavandulol, or artemisia alcohol are less common and outside the scope of this review, in which we focus on linalool, geraniol and their oxygenated derivatives.

Monoterpenols differ from other aliphatic monoterpenes in their chemical properties. The presence of an alcohol functional group makes them not only more polar and, therefore, soluble in water (Weidenhamer et al., 1993), but also more chemically reactive. They are, in addition, reported to isomerize to one another in acidic aqueous solvents (Baxter et al., 1978; Cori et al., 1986), although these harsh conditions are probably not achieved in biological systems. The alcohol functional group also makes monoterpenols more amenable to secondary transformations, such as oxidation, glycosylation (Sheth et al., 1961; Inouye et al., 1963; Francis and Allcock, 1969), esterification (Steltenkamp and Casazza, 1967) or methylation (Froehlich et al., 1989). Among these transformations, glycosylation is the most ubiquitous: many plants store monoterpenols as water-soluble glycosides. Plants presumably glycosylate monoterpenols to decrease their toxicity and facilitate their storage or transport between plant organs (Vasserot et al., 1995). In this review, we focus on the recent advances in the understanding of monoterpenol oxidative metabolism that greatly diversifies the structures of derived compounds and changes their chemical properties. This diversification is reflected in the different roles of the resulting products, as well as their different economical uses.

## Ecological and Economic Importance of Monoterpenol Derivatives

### Iridoids Are Geraniol Derivatives with Important Medicinal Properties

Only few oxygenated linear geraniol derivatives have been reported in plants. Those include 8-hydroxygeraniol, foliamenthoic acid and carboxygeranic acid, conjugated to hexose, pentose, malonyl, or acetyl groups. These compounds were detected upon expression of cytochromes P450 CYP76C4 and CYP76B6 in *Nicotiana benthamiana* (Höfer et al., 2013), but their biological relevance and occurrence *in vivo* has not yet been demonstrated.

**FIGURE 2:**
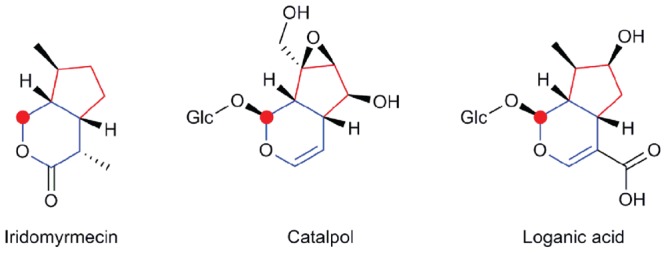
**Examples of iridoids.** Iridomyrmecin is found in ants (Iridomyrmex sp), catalpol and loganic acid are found in various plants. The cyclopentane and pyran rings are colored in red and blue, respectively. Carbon-1, on which glycosylation commonly takes place is marked by a red circle. Glc, (β -D)-glucopyranosyl moiety.

The best documented oxidized derivatives of geraniol are iridoids. Those are cyclic derivatives, with a fused cyclopentane and pyran ring system potentially decorated at various positions and glucosylated at position C-1 (**Figure 2**). In plants, iridoids are mostly found as conjugates (iridoid glycosides or IGs). They were first isolated in the mid-1800s from the root of *Rubia tinctorum* (Schunck, 1848), and were named according to their similarity of structure and biosynthetic origin to iridodial and iridomyrmecin found in the ants of the genus *Iridomyrmex* (Cavill et al., 1956; Bowers, 1991). Iridoids are found in more than 57 dicot families (Bowers, 1991) that belong to Asteridae, such as Apocynaceae, Rubiaceae, Lamiaceae, Loganiaceae, Verbenaceae, Valerianaceae, Gentianaceae, or Scrophulariacea. Because of their distribution and diversity, they can be used as chemotaxonomic markers. Iridoid producing plants have been used in folk medicine for the treatment of many diseases such as parasitoses, inflammation, diabetes, and others (Chan-Bacab and Peña-Rodríguez, 2001; Viljoen et al., 2012).

**FIGURE 3:**
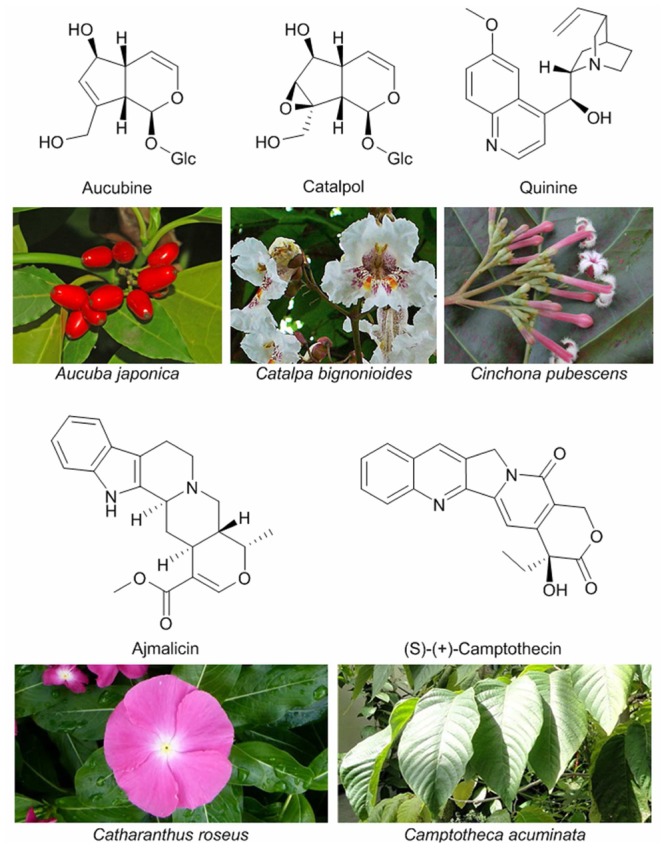
**Examples of iridoids (aucubin and catalpol) and iridoid-derived alkaloids, and the plants producing them.** Photos by Hectonichus/CC BY-SA 3.0 (*Aucuba japonica*), H. Zell/GFDL 2.0 (*Catalpa bignonioides*), Forest and Kim Starr/CC BY 3.0 (*Cinchona pubescens*), Wikimedia Commons (*Catharanthus roseus* and *Camptotheca acuminata*).

The number of plant iridoids is estimated to reach 3000 molecules according to a thorough literature-based inventory performed by Dinda et al. (2007a,b, 2009, 2011), and, due to their proven or potential pharmacological properties, the number of studies on these molecules is rising quickly. Many iridoids and iridoid-derived compounds, such as monoterpene indole alkaloids (MIAs), are used in modern medicine for their antibacterial, anti-inflammatory, anti-tumoral, chemopreventive and various others activities (Ghisalberti, 1998; Dinda and Debnath, 2013). Among this variety of molecules, vindoline and vincristine, extracted from Madagascar periwinkle (*Catharanthus roseus*) are used directly or as precursors for the synthesis of drugs for treatment of lung cancer and lymphoma. Other extensively used MIAs include compounds as diverse as the antitumoral camptothecin (Wall et al., 1966), the antihypertensive ajmalicin or the classical antimalarial drug quinine (Block, 1999; **Figure 3**). This review focuses on the role and origin of monoterpenol iridoids in plants. The description of their medicinal properties can be found in dedicated reviews (Ghisalberti, 1998; Dinda and Debnath, 2013).

Iridoids appear to accumulate in plants as a protection against various herbivores and pathogens. A toxic effect of isolated compounds against vertebrate and invertebrate predators has been reported (Bowers and Puttick, 1988; Puttick and Bowers, 1988; Bowers, 1991). They also have antibacterial (Rombouts and Links, 1956; Ishiguro et al., 1982; Davini et al., 1986) and antifungal (Sluis et al., 1983; Davini et al., 1986; Marak et al., 2002; Biere et al., 2004) activities. The toxic effect of the IGs on invertebrates and microorganisms is due to the activity of their aglycone moiety, which is released through enzymatic or non-enzymatic acidic hydrolysis by plant or insect β-glucosidases (Baden and Dobler, 2009; Dobler et al., 2011). In some cases, the inhibitory effect of IGs on insects was only observed after a first cleavage of IG by β-glucosidases, which led to the release of the toxic aglycone (Sluis et al., 1983; Marak et al., 2002). Cleavage can also release the IG from a more complex structure, For example, a β-glucosidase from the leaves of *Ligustrum obtusifolium* has been demonstrated to convert the secoiridoid glucoside moiety of oleuropein into a glutaraldehyde-like structure with strong protein denaturing, protein crosslinking, and lysine-alkylating activities (Konno et al., 1999).

It was recently suggested that IGs could also have a role during oxidative stress, such as observed upon drought conditions. Drought stress increases the accumulation of the IGs catalpol, aucubin, harpagide, and harpagoside in roots of the medicinal plant *Scrophularia ningpoensis* (Wang D. et al., 2010). Based on the protective activities of catalpol and aucubin observed in animal studies (Jin et al., 2008), it was suggested that this increase in IGs content may help the plant cell to deal with oxidative stress (Mao et al., 2007). An increase in indole alkaloid concentrations and particularly of the antioxidant alkaloid ajmalicin in *C. roseus* plants submitted to drought stress has also been reported (Jaleel et al., 2008a,b).

The toxic effect of iridoids toward insects is not general and has to be examined from a co-evolutionary point of view. They can have a clear deterrent effect for non-adapted insects, but can be phagostimulants for adapted ones (Dobler, 2001). Feeding generalist insects with iridoid glycosides reduces growth rate, increases larval stages duration, and decreases survival rates (Bowers and Puttick, 1988, 1989; Puttick and Bowers, 1988). As for the deterrent effect, it is still not clear if it is due to the glycosides or to the release of the aglycones via acid-hydrolysis or insect-derived β-glycosidases in the insect midgut (Marak et al., 2002). Contrary to generalists, adapted insects can feed on iridoid-producing plants, even with beneficial effects. Iridoids can act as oviposition stimulants (Pereyra and Bowers, 1988; Prudic et al., 2005) and feeding stimulants (Bowers, 1983) for both adults and larvae. The iridoid resistance of specialist insects can be conferred by the ability to absorb and substract them from the gut before hydrolysis can occur (Dobler et al., 2011). The absorbed iridoids can either be degraded, as observed in noctuid and geometrid larvae (Boros et al., 1991), or sequestrated as observed in many adapted Coleoptera, Homoptera, Hymenoptera, or Lepidoptera, thereby conferring those insects obvious advantages against herbivores, parasites, or pathogens (Gardner and Stermitz, 1988; Rimpler, 1991; Bowers and Stamp, 1997). Only a few iridoids, such as aucubin and catalpol, are sequestered, suggesting either highly specific transport mechanisms from the insects gut or a differential degradation process between compounds (Dobler et al., 2011). Other insects have been shown to exploit the protective iridoid properties through *de novo* biosynthesis, as observed in some species of chrysomelines (Kunert et al., 2008). *De novo* synthetized iridoids can constitute a part of the defensive chemical arsenal of insects, and they can also serve as a tightly regulated sex pheromone signal, as observed in some species of aphids (Dawson et al., 1990; Stewart-Jones et al., 2007).

### Monoterpenol Derivatives in Food and Beverage Aroma

**FIGURE 4:**
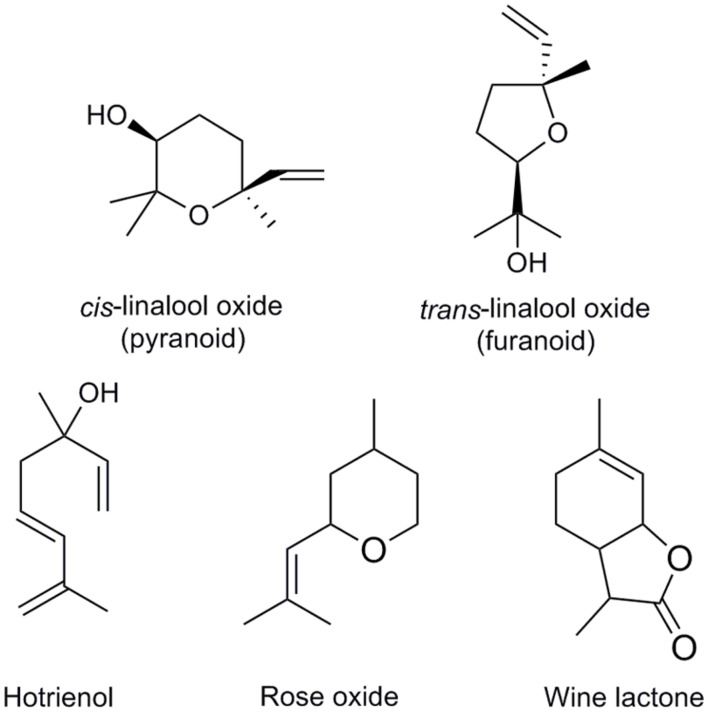
**Selected monoterpenol derivatives that occur in food and beverages**.

Development of gas chromatography in the 1950s (James and Martin, 1952) triggered interest for the analysis of volatile constituents of food, beverages, and aromatic plants. These included many putative oxygenated linalool derivatives, but they were always found in complex mixtures making unambiguous identification impossible. In 1963 a method for synthesis of 6,7-epoxylinalool was reported (Felix et al., 1963). In acid, this epoxide could be further transfomed to form furanic or pyranic linalool oxides *cis* or *trans* diastereomers (structures with 5- or 6-membered ring, respectively, **Figure 4**). In addition, each of these linalool oxide diastereomers exists as two enantiomers, giving a total of 8 stereoisomers. Interestingly, pairs of enantiomers have different sensory properties, depending on whether they formed from (*S*)- or (*R*)-linalool. Linalool oxides derived from (*S*)-linalool are described as sweet, floral and creamy, and those derived from (*R*)-linalool as earthy or leafy (Wang et al., 1994). Availability of synthetic reference standards enabled identification of linalool oxides in green and black tea (Yamanishi et al., 1964). After this pioneering work on tea aroma, linalool oxides were discovered in many other fruits and drinks (**Table 1**). Other oxygenated linalool derivatives were later discovered in other plants, such as 6- and 7-hydroxylinalool in camphor tree (*Cinnamomum camphora*; Takaoka and Hiroi, 1976). Analysis of glycosylated volatiles allowed for identification of additional monoterpenol derivatives, such as 8- and 9-hydroxylinalool in birch (*Betula alba*) and Japanese quince (*Chaenomeles japonica*; Tschesche et al., 1977).

**Table 1:** Monoterpenol derivatives involved in flavor and fragrance of fruits and other agriculturally important plant species.

Plant	5-hydroxylinalool	6-hydroxylinalool	7-hydroxylinalool	6,7-epoxylinalool	6,7-dihydroxylinalool	8-hydroxylinalool	9-hydroxylinalool	8-carboxylinalool	Wine lactone	*trans-*linalool oxide (5)	*cis*-linalool oxide (5)	*trans*-linalool oxide (6)	*cis*-linalool oxide (6)	Anhydrolinalool oxide (5)	Hotrienol	7-hydroxydihydrolinalool	Rose oxide	Reference
*Actinidia arguta* (kiwi)			B			B	B											Garcia et al., 2011
*Camellia sinensis* (tea)										F B	F B	F B	F B					Bondarovich et al., 1967; Moon et al., 1996; Wang et al., 2000
*Carica papaya* (papaya)		F	F B	F	F B	F B	F B			F B	F B	F	F		B			Flath and Forrey, 1977; Winterhalter et al., 1986
*Cichorium endivia* (endive)										F	F							Goetz-Schmidt and Schreier, 1986
*Citrus paradisi* (grapefruit)									F									Buettner and Schieberle, 1999
*Coffea arabica* (coffee)										F	F			F				Gautschi et al., 1967
*Cymbopogon* (citronelle)																	F	Kreis and Mosandl, 1994
*Ficus carica* (fig)										F	F	F	F					Gibernau et al., 1997
*Solanum lycopersicum* (tomato)										B	B	F			B			Buttery et al., 1971, 1990
*Mangifera indica* (mango)						F B				B	B							Sakho et al., 1997; Ollé et al., 1998
*Citrus sinensis (orange*)						F			F	F								Hinterholzer and Schieberle, 1998; Selli et al., 2004
*Passiflora* (passionfruit)		B	B			B	B											Chassagne et al., 1999
*Prunus armeniaca* (apricot)		B				B				B	B	B	B			B		Krammer et al., 1991; Salles et al., 1991
*Prunus domestica* (yellow plum)						B												Krammer et al., 1991
*Prunus persica* (peach)						B						B	B					Krammer et al., 1991
*Ribes nigrum* (blackcurrant*)*										F B							F	Varming et al., 2006
*Vitis vinifera* (grape)	B	F B	F B		F	B		B		F B	F B	F B	F B		F		F	Williams et al., 1980, 1981; Wilson et al., 1984; Strauss et al., 1988; Guth, 1997; Winterhalter et al., 1997
*Zingiber officinale* (ginger)						B												Wu et al., 1990

*All compounds derive from linalool except for rose oxide that derives from citronellol.F, free; B, bound.*

Studies of grape (*Vitis vinifera*) volatiles revealed a particularly rich linalool metabolism. Some of linalool diols in grape juices are unstable in acidic solutions and can spontaneously form more stable structures by cyclisation or elimination of a water molecule (Williams et al., 1980). These rearrangements can be accelerated by heating. The products of these rearrangements are more volatile and can have a stronger odor compared to their precursors: for example, hotrienol (**Figure 4**), which forms from relatively odorless 7-hydroxylinalool upon acidification, has sweet tropical scent. Similarly, the acid 8-carboxylinalool was shown to cyclize in acidic medium to form a potent sweet and coconut-like odorant wine lactone (Bonnländer et al., 1998; **Figure 4**). This reaction is thought to occur during wine aging. Rose oxide is another monoterpenol derivative with a strong rose-like aroma, which was first discovered in rose flowers (Seidel et al., 1961), and later as a constituent of fruits and other food ingredients (**Table 1**). Rose oxide differs from other compounds described above in that it is not derived from linalool, but citronellol, a reduced geraniol/nerol derivative.

Oxygenated monoterpenol derivatives contribute to characteristic aroma of other plants, among them many agricultural crops (**Table 1**). Some have a weaker odor, but can rearrange in acidic medium and elevated temperatures. They might act as precursor of flavor generated in foods and beverages during processing or storage. Similarly, glycosides can act as flavor precursors and release their volatile aglycones during food or beverage preparation, fermentation or storage.

### Linalool and its Derivatives in Plant–Insect Interactions

Linalool has been extensively investigated for its role in plant-insect interactions, including pollinator attraction (Borg-Karlson et al., 1996; Raguso and Pichersky, 1999; Reisenman et al., 2010), defense (Junker et al., 2011; McCallum et al., 2011; Xiao et al., 2012), and involvement in multi-trophic interaction by attraction of herbivory predators and parasites (Loughrin et al., 1995; Turlings et al., 1995; Xiao et al., 2012). However, the role of a large number of compounds deriving from linalool oxidative metabolism remains elusive. A few studies showed that pyranoid and furanoid linalool oxides from *Clarkia breweri* (Raguso and Pichersky, 1999) and *Daphne mezereum* (Borg-Karlson et al., 1996), as well as lilac compounds in *Silene latifolia* (Dötterl et al., 2006a,b, 2007) are important olfactory cues for pollinators. For example, the moth *Hadena bicruris* specifically recognizes lilac aldehydes emitted by *S. latifolia* at night among the many volatiles of the scent bouquet in a nursery pollination system. The moth then lays its eggs in the flower and pollinates it at the same time (Dötterl et al., 2006a,b, 2007). In another example, a link was established between the emission level of lilac aldehydes and attractiveness of the plants from the *Asimitellaria* lineage for different pollinators, namely short- and long-tongued fungus gnat (Okamoto et al., 2015). Olfactometer trials demonstrated that lilac aldehydes induced nectaring behavior of the long-tongued fungus gnats, but repelled short-tongued fungus gnats. The volatile composition of several *Asimitellaria* species is adapted to their specific pollinator species of fungus gnat, illustrating the scent-mediated speciation of *Asimitellaria* to their pollinators. Moreover, pure lilac aldehydes and lilac alcohols were shown to repel thrips and hoverflies (Boachon et al., 2015), suggesting that linalool and its derivatives have a dual role in attraction of beneficial and repellence of neutral or detrimental insects. The need for cross-pollination or its absence may determine the balance between these two roles. For example, *Arabidopsis thaliana* flowers do not need pollinators to reproduce, although out-crossing events have been observed in natural populations since flowers are visited by insects such as solitary bees, hoverflies, and thrips (Jones, 1971; Snape and Lawrence, 1971; Hoffmann et al., 2003). As a consequence, *A. thaliana* chemical profile may favor protective functions, such as defense against flower visitors and pollen thieves, over pollinator attraction.

In addition to its role in scent production and plant-insect interactions, linalool oxidative metabolism in plants might serve for linalool detoxification as is the case in insect guts (Yu, 1987; Southwell et al., 1995), fungus *Botrytis cinerea* (Bock et al., 1986) and soil fungi (Demyttenaere and Willemen, 1998). Local metabolism of linalool into its furanoid and pyranoid oxides in pistils of *C. breweri* was proposed as a defense mechanism to protect pollen tube from linalool toxicity (Raguso and Pichersky, 1999). Oxidation and subsequent glycosylation also increases the solubility of monoterpenols, and may favor both their sequestration and transport to other organs. Their presence in phloem might serve as a line of defense against phloem-feeding insects (Gowan et al., 1995).

## First Insights into Geraniol and Linalool Oxidative Metabolism

Battersby et al. (1966) first demonstrated with labeling experiments that geraniol was cyclized and then transformed to loganin on the pathways leading to MIAs. Since then, efforts have been devoted to understanding the steps from geraniol to the common MIA precursor secologanin, as well as other species-specific downstream alkaloids. The Damtoft and Inouye groups paved the way in the 1980s (Damtoft et al., 1981, 1983; Uesato et al., 1984a,b; Inouye and Uesato, 1986), with extensive precursor feeding experiments in which the patterns of labeled carbon scrambling were studied in many plants, thus asserting the different iridoid pathways leading from the monoterpenol geraniol (and citronellol in a few cases) to iridodial and alkaloids (reviewed in details by Jensen, 1991). In particular, they demonstrated that geraniol was the precursor of most of the iridoids, and established that deoxyloganic and epi-deoxyloganic acids were crucial intermediates in the synthesis of MIAs, each present in specific pathways. The putative pathway leading to these compounds was predicted to involve a succession of oxido-reductions, but until recently the exact sequence and most of the enzymes involved remained unknown.

Active linalool oxidative metabolism in plants was initially observed when feeding linalool to tobacco cell cultures, which led to the production 8-hydroxylinalool (Hirata et al., 1981). In Clarkia, both linalool and linalool oxides were found emitted from flowers, with linalool oxides likely to be formed in specific tissues from an enzymatic linalool oxidation pathway (Pichersky et al., 1994; Raguso and Pichersky, 1999). The existence of this oxidative metabolism in plants was further supported by the detection of an increased production of both volatile and soluble oxygenated linalool derivatives upon heterologous expression of linalool synthases in different plant species, including tomato, carnation and *A. thaliana* (Lewinsohn et al., 2001; Lavy et al., 2002; Aharoni et al., 2003).

The biosynthetic route and fate of linalool in the plant were then more thoroughly investigated using labeled precursors. The first studies, carried out with lilac flowers using both deuterated and ^18^O labeled precursors indicated that lilac compounds derived from linalool via a plastidial (MEP) pathway and proceeded with low enantioselectivity, but with high conservation of the (*R*) or (*S*) configuration of the linalool precursor (Burkhardt and Mosandl, 2003; Kreck et al., 2003). The pathway appeared to sequentially involve 8-hydroxylinalool, 8-oxolinalool, and lilac aldehydes converted into lilac alcohols. Interestingly, labeled lilac compounds were found associated with the plastids only when feeding was performed on intact plant tissues, but not isolated plastids. Linalool feeding experiments in kiwi (*Actinidia arguta*) flowers revealed the formation of lilac alcohol epoxides (Matich et al., 2006). (*S*)-linalool in kiwi flower petals was also shown to be stereospecifically converted to lilac compounds (Matich et al., 2011).

In grape berries, the complexity of linalool metabolism was explored by Luan et al. (2006) via feeding labeled linalool analogs. This investigation revealed a linalool oxidative metabolism leading to linalool oxides, 8-hydroxylinalool, 7-hydroxylinalool, 6-hydroxylinalool, hotrienol and nerol oxide, as well as their glycoconjugates. Hydroxylation at the 7 position was stereoselective, which strongly suggested that it results from an enzymatic and not a photooxidation reaction. This work also demonstrated that linalool oxides were preferentially formed via a 6,7-epoxylinalool intermediate, and also to a minor extent through 6,7-dihydroxylinalool, thus most likely via an enzymatic reaction. It showed, in addition, that the total oxygenation activity was stronger at the beginning of the ripening period, and that stereospecific formation of furanoid and pyranoid linalool oxides occurred at different stages of the berry maturation. Interestingly, the same group also investigated the metabolism of deuterated geraniol in grape, but hydroxylated geraniol derivatives could not be detected (Luan et al., 2005). Some geraniol was, however, converted to rose oxide, a cyclic ether reported to be derived from oxidative metabolism of citronellol, a reduced derivative of geraniol in grape (Luan et al., 2005). It thus appears that geraniol is mostly oxidized at the terminal positions and subsequently turned into iridodial and epi-iridodial, and besides terminal aldehydes and carboxygeraniol, few other natural chain hydroxylated or oxygenated derivatives are reported. Conversely, oxidation of linalool occurs at different positions in the carbon chain forming a wide diversity of hydroxylated compounds.

## Recent Advances and Role of Cytochromes P450 in Geraniol and Linalool Metabolism

### Geraniol Metabolism in *C. roseus*

Starting in the early 1970s, geraniol and nerol were reported to be almost exclusively oxidized at the C8 position by a monooxygenase activity present in *C. roseus* (or *Vinca rosea*) extracts, to form 8-hydroxygeraniol (then referred as 10-hydroxygeraniol; Meehan and Coscia, 1973; Madyastha et al., 1976). Twenty years later, the first plant P450 characterized at the molecular level, CYP71A1, was isolated from the avocado fruit, and shown to catalyze the 2,3- or 6,7-epoxidation of geraniol and nerol *in vitro* (Hallahan et al., 1992). A homolog CYP71 from catmint was then found to catalyze the 8-hydroxylation of geraniol and nerol (Hallahan et al., 1994). However, in both cases, no further experiment confirmed the physiological function of these enzymes *in planta*. More focused investigations, targeting the first oxidative step in the pathway leading from geraniol to MIAs, were required to characterize CYP76B6 as a geraniol hydroxylase in *C. roseus,* and this activity was found to be related to the accumulation of alkaloids in Apocynaceae (Collu et al., 2001). A more thorough functional analysis of CYP76B6 catalytic properties only recently demonstrated that this P450 in fact catalyzes two successive regio-specific oxidations at the C8 position of geraniol to form the derived aldehyde (Höfer et al., 2013). The strong induction of the *CYP76B6* gene expression by the hormone methyl-jasmonate, as well as its co-expression with the other MIA genes in the phloem-associated parenchyma confirmed that it is the best candidate for catalyzing the geraniol oxidation step toward iridoids and MIAs in *C. roseus* (Miettinen et al., 2014). Similarly, in the iridoid producing plant *Swertia mussotii*, the methyl jasmonate-induced CYP76B10 was found to catalyze the 8-hydroxylation of geraniol (Wang J.F. et al., 2010). This activity is likely widespread in the plant kingdom beyond Asteraceae and iridoid-producing plants, as similar oxidized geraniol derivatives have been reported in tobacco (a plant that does not produce geraniol) upon expression of geraniol synthases (Dong et al., 2013; Höfer et al., 2013). Accordingly, other members of the CYP76 family were found to catalyze oxidation of geraniol or nerol such as CYP76C4 from *A. thaliana*, catalyzing both the geraniol to 8- and 9-hydroxylations, and CYP76B1 from *Helianthus tuberosus*, catalyzing nerol hydroxylation (Höfer et al., 2013). CYP76s from *A. thaliana* were also shown to metabolize other monoterpenols. This suggested that other members of the CYP76 family might be involved in monoterpenol metabolism.

**FIGURE 5:**
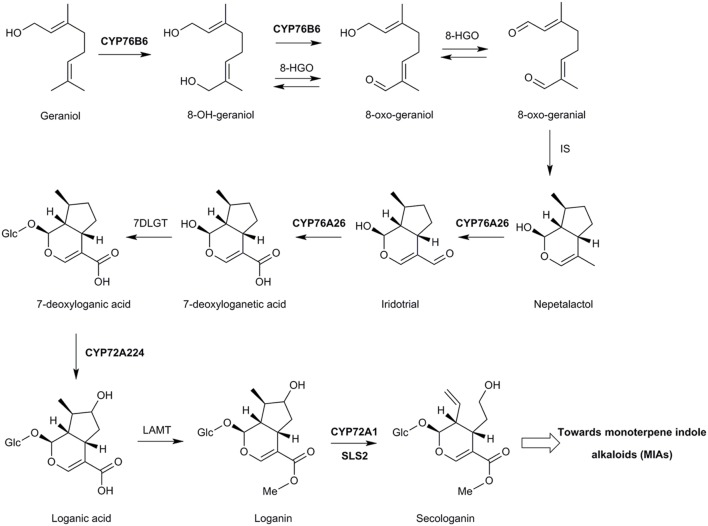
**The pathway leading from geraniol to secologanin in *C. roseus*.** CYP76B6: Geraniol 8-oxidase (Collu et al., 2001; Höfer et al., 2013; Miettinen et al., 2014), 8-HGO: 8-hydroxygeraniol oxidoreductase (Miettinen et al., 2014), IS: iridoid synthase (Geu-Flores et al., 2012). 7DLGT: 7-deoxyloganetic acid glycosyltransferase (Asada et al., 2013; Miettinen et al., 2014), LAMT: loganic acid methyltransferase (Madyastha et al., 1973; Murata et al., 2008), CYP72A1: secologanine synthase (Irmler et al., 2000), SLS2, secologanine synthase 2 (de Bernonville et al., 2015). Glc, (β-D)-glucopyranosyl moiety.

This was confirmed when deep-sequencing and proteomic approaches led to the elucidation of the complete secologanin pathway in *C. roseus* (**Figure 5**). Studies simultaneously carried out in different laboratories led to the characterization of CYP76A26 from *C. roseus* as a nepetalactol (iridodial) oxygenase, catalyzing the three consecutive oxygenation steps forming 7-deoxyloganic acid (Salim et al., 2013; Miettinen et al., 2014). Interestingly, this enzyme was also found capable to hydroxylate monoterpenols (such as nerol, citronellol), although with low efficiency. CYP76A26, however, did not hydroxylate geraniol, possibly preventing competition with CYP76B6. The same or similar strategies led the characterization of the complete sequence of enzymes forming the secologanin pathway (**Figure 5**). Those include one or possibly redundant oxidoreductase(s) (8-hydroxygeraniol oxidoreductase, 8-HGO) converting 8-oxogeraniol to 8-oxogeranial thus having a partially redundant role with CYP76B6 (Miettinen et al., 2014; Brown et al., 2015; Krithika et al., 2015), an iridoid synthase (IS) belonging to the progesterone 5-β reductase family among which this activity seems widespread (Geu-Flores et al., 2012; Munkert et al., 2015), a glycosyltransferase (7-deoxyloganic acid glycosyltransferase, 7-DLGT), and another cytochrome P450, CYP72A224, to form loganic acid (Miettinen et al., 2014; Salim et al., 2014). Interestingly, an homolog of 8-HGO, CYPADH, was recently shown to improve the flow of the pathway in engineered yeast, most probably by facilitating the reaction catalyzed by CYP72A224, i.e., the formation of loganic acid (Brown et al., 2015).

The final steps of methylation and oxidative ring opening by loganic acid methyltransferase (LAMT) and secologanin synthase (SLS1 or CYP72A1), respectively, had been described previously (Madyastha et al., 1973; Irmler et al., 2000; Murata et al., 2008). Recently, a second functional *C. roseus* secologanin synthase, SLS2, was characterized and both isoforms were shown to further catalyze the oxidation of secologanin to secoxyloganin *in vitro* (de Bernonville et al., 2015).

It is interesting to note that both CYP72A1 and CYP72A224 share the unusual property of metabolizing larger and very hydrophilic glycosylated substrates. CYP72A224 was confirmed to be inactive on the aglycone (Miettinen et al., 2014; **Figure 5**), which was not tested for SLS1 and SLS2. The same sequence of two reactions, i.e., glycosylation then hydroxylation, was also reported for the related geniposide pathway in *Gardenia jasminoides* (Nagatoshi et al., 2011). It is thus likely that CYP72s derive from a CYP ancestor that was already able to accommodate substrates larger than monoterpenols. This hypothesis is supported by the hydroxylation of triterpenic derivatives by CYP72s in different species such as *Medicago truncatula* (Biazzi et al., 2015) or the quinidine hydroxylation by CYP72A8 from *A. thaliana* (Olry et al., 2007).

Other cytochromes P450 have been identified in biosynthetic pathways from geraniol to MIAs. Twenty years ago, CYP71D12 was characterized in *C. roseus* as a tabersonine hydroxylase catalyzing the first committed step to vindoline (St-Pierre and De Luca, 1995), and recently, CYP71D1v2 was shown to also act in the pathway as a tabersonine 3-oxygenase (Qu et al., 2015) catalyzing the formation of a 2,3-epoxide, which can undergo rearrangement to yield the vincamine-eburnamine backbone (Kellner et al., 2015). Considering the wide diversity of MIAs, it seems reasonable to expect that more P450 oxygenases still remain to be characterized in their respective pathways.

### Linalool Metabolism in *A. thaliana* Flowers

**FIGURE 6:**
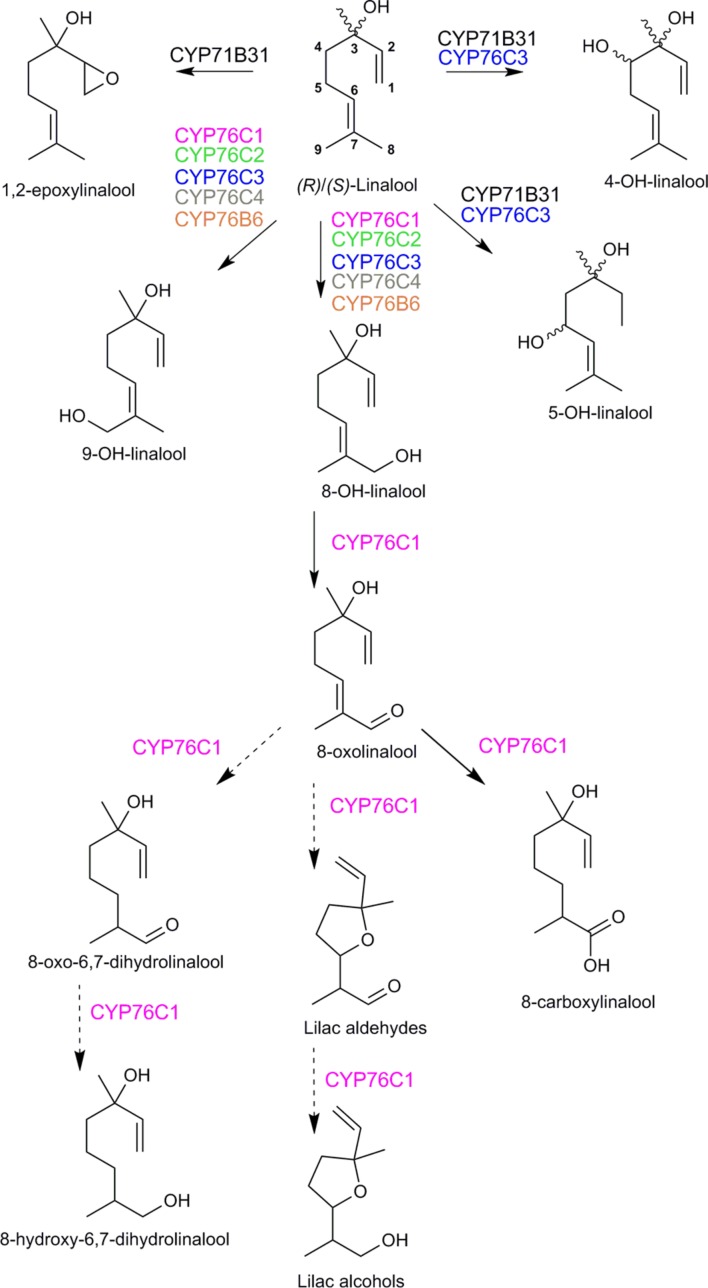
**Linalool metabolism by *Arabidopsis thaliana* cytochromes P450.** Activities were validated in yeast or *Nicotiana benthamiana* leaves. Dotted arrows indicate reactions that may involve other enzymes. CYP71B31, CYP76C1, and CYP76C3 metabolized both (*R*)- and (*S*)-linalool, the enantiomers were not individually tested as substrates for the other CYP76 enzymes. The stereochemistry of the products is indicated only when it was determined. A complex mixture of lilac alcohol and lilac aldehyde diastereoisomers was obtained (in unequal amounts, but too low for proper quantification). According to Ginglinger et al. (2013), Höfer et al. (2014), Boachon et al. (2015).

*A. thaliana* flowers were long considered as scentless and a poor model to study the metabolism of volatile compounds. However, head-space analyses revealed that whereas sesquiterpenoids were predominant in the floral bouquet, small amounts of linalool and lilac aldehydes could also be detected (Chen et al., 2003; Rohloff and Bones, 2005; Tholl et al., 2005). In addition, Chen et al. (2003) reported a flower-expressed terpene synthase (TPS14; At1g61680) producing (*S*)-linalool. The first lead to linalool oxygenases emerged from a systematic *in silico* analysis of gene co-expression (Ehlting et al., 2008). Two P450 genes *CYP76C3* and *CYB71B31* appeared tightly co-expressed in flowers with the genes encoding two terpene synthases *TPS10* and *TPS14* characterized as (*R*)- and (*S*)-linalool synthases, respectively (Ginglinger et al., 2013). Both P450s were expressed exclusively in the upper segment of the anther filaments and nectaries (and weakly in petals) upon anthesis. Both P450s could metabolize both linalool enantiomers in yeast and after transient expression in *N. benthamiana*. CYP71B31 formed 1,2-epoxylinalool and a mixture of different diastereoisomers of 4- and 5-hydroxylinalool, and CYP76C3 generated 8- and 9-hydroxylinalool and a different mixture of 4- and 5-hydroxylinalool diastereoisomers (**Figure 6**). However, altered expression of CYP76C3 and CYP71B31 in insertion mutants had only a minor impact on the overall floral linalool oxidative metabolism, which prevented the identification of their end-chain endogenous products, and suggested the contribution of other enzymes to the floral linalool metabolism.

The other members of the CYP76 family in *A. thaliana* were thus more extensively investigated. At first, their enzyme activity was tested *in vitro* together with the activity of CYP76B6 from *C. roseus* and CYP76B1 from *H. tuberosus* (Höfer et al., 2014). All the enzymes that could be successfully expressed in yeast metabolized monoterpenols, but only CYP76C1, CYP76C2, CYP76C4, and CYP76B6 metabolized linalool into 8-hydroxylinalool as major product and 9-hydroxylinalool as minor product. In addition, CYP76C2 and CYP76C4 were shown to produce 1,2-epoxylinalool (**Figure 6**). This suggested a widespread monoterpenol oxidation capacity of the CYP76 family, and especially a linalool oxidase role of the CYP76C subfamily in Brassicaceae (Höfer et al., 2014).

From all the *in vitro* tested enzymes, CYP76C1 was by far the most efficient linalool-converting enzyme, appearing as a prime linalool oxidase candidate in *A. thaliana*. Its enzyme activity and role in the plant were thus extensively investigated (Boachon et al., 2015). Like *CYP71B31* and *CYP76C3*, *CYP76C1* was co-expressed with *TPS10* and *TPS14* upon flower anthesis, but *CYP76C1_promoter_:GUS* transformants revealed a more widespread expression in flower organs, including anthers, stigma and petals. *In vitro* studies, transient expression in *N. benthamiana,* as well as targeted metabolic profiling in *Arabidopsis* mutants altered in *CYP76C1* expression all confirmed that CYP76C1 catalyzed sequential oxidation of the terminal 8 carbon of linalool, forming successively 8-hydroxylinalool, 8-oxolinalool, and 8-carboxylinalool (**Figure 6**). Furthermore, 8-oxolinalool was converted by CYP76C1 into the lilac aldehydes and lilac alcohols in the *in vitro* assays (**Figure 6**). All products, except for 8-oxolinalool, were detected as volatile and conjugated compounds in flowers and their amount was dependent on *CYP76C1* expression. The high linalool oxidase activity of CYP76C1 thus most likely explains the minor quantitative impact of CYP76C3 and CYP71B31 *in vivo* (our group, unpublished data). The specific reactions catalyzed by CYP71B31 and CYP76C3, as well as their tissue-specific expression, suggest the existence of a complex linalool metabolism in *A. thaliana* flowers for the production of specific cues or protection compounds. In the same line, additional linalool derivatives, such as 8-hydroxy-6,7-dihydrolinalool and 8-oxo-6,7-dihydrolinalool, although formed *in vitro* are only barely altered or not detected in the flowers of the *cyp76c1* line. Since none of the P450s investigated was able to catalyze the production of such compounds *in vivo*, it is likely that other linalool-metabolizing enzymes are present in *A. thaliana*.

Interestingly, subcellular localization experiments performed with the *A. thaliana* CYP76s provided a possible explanation for the initial observations of Kreck et al. (2003), who found that labeled lilac compounds associated with the plastids only when feeding was performed on intact plant tissues, but not isolated plastids. The subcellular localization of CYP76C1, CYP76C3, and CYP71B31 was investigated in parallel with the localization of TPS10 and TPS14 (Ginglinger et al., 2013; Boachon et al., 2015). The two linalool synthases were detected in vesicular structures associated with the plastids, when the P450 proteins appeared associated with plastid-wrapping endoplasmic reticulum (ER) sheets. The lilac compounds detected in intact tissues may thus result from this plastid-ER organization.

The availability of *CYP76C1* mutants also offered the opportunity to investigate the role of the products of linalool oxidative metabolism in ecological interactions with the flowers of *A. thaliana* (Boachon et al., 2015). The depletion of volatile and soluble linalool metabolites rendered the *cyp76c1* inactivation mutant flowers more attractive and susceptible to several antagonist insects, such as the pollen thieves thrips (*Frankliniella occidentalis*) and florivores, including a generalist herbivore *Plutella xylostella* and the Brassicaceae specialists*, Phaedon cochleariae* and *Spodoptera littoralis*. Accordingly, the beetle *P. cochleariae* was shown to prefer feeding on control cabbage leaves rather than leaves treated with 8-hydroxylinalool or 8-carboxylinalool. Since plant linalool oxides are mainly stored as glycosides, they could thus function as toxic compounds or deterrents released from wounded tissues or in the insect guts upon herbivory attack. In addition, CYP76C1 produced volatile lilac aldehydes and alcohols with a simultaneous decrease in the emission of their precursor, linalool. Since thrips and *A. thaliana* pollinator hoverfly (*Episyrphus balteus*) are attracted by linalool and repelled by both lilac aldehydes and lilac alcohols, CYP76C1 appears to set the balance between an attractive display and defense, by consuming linalool and producing repellant lilac compounds. In good agreement with this hypothesis, *CYP76C1* is a pseudogene in the genome of the obligate outcrossing relative *Arabidopsis lyrata* (Höfer et al., 2014). Altogether, this suggests that CYP76C1, responsible for the synthesis of most oxygenated linalool derivatives in *A. thaliana*, may increase flower fitness through defense against floral antagonists.

## Perspectives and Open Questions

Considering their broad occurrence and economic importance, monoterpenols and their derivatives are prime targets for pathway discovery in higher plants. Recent work started to reveal the core monoterpenol oxidative metabolism. Advances in the understanding of linalool and geraniol oxidative pathways occurred simultaneously, revealing some similarities, but also differences possibly resulting from the intrinsic properties of each compound. Linalool and geraniol metabolism share common initial oxidation steps. The most common oxidation for both of them occurs at the most stable 8-carbon allylic position, and involves several consecutive oxygenation steps leading to the formation of the derived alcohol, of the aldehyde, and with the acid as a final product, although some enzymes form only the alcohol or the aldehyde intermediates. Both the alcohol and the acid are usually accumulated in plants as glycosides. The detection of conjugated intermediates and the existence of branching pathways suggest that each intermediate is released from the oxygenases. Not surprisingly, the most polar derivatives, such as carboxylic acids and glycosides have been long overlooked due to analytical procedures essentially restricted to GC. This points to the importance of using complementary analytical approaches to get the full appraisal of terpene-derived metabolic pathways.

In all cases reported so far, the initial oxidation steps were catalyzed by cytochromes P450 from the CYP76 family. The enzymes catalyzing this initial monoterpenol oxidation belong to different CYP76 subfamilies in different plant taxa, and thus share quite low sequence identities (<55%). The CYP76 family proteins and functions thus do not seem to be well fixed, but significantly vary in plant lineages, while maintaining common properties. The main branching point in the oxidation cascade is usually the aldehyde intermediate. All the intermediates can be glycosylated at the carbon position oxidized by the P450 enzyme, except the aldehyde, which is also most often not accumulating or not observed in plants, possibly due to its quick processing or its intrinsic reactivity. The balance between the glycosyltransferase activities and the efficiency of the different oxidation steps seems to control the flux through the pathway, and, consequently, the distribution among the different products.

In Asteridae, the elongation of the geraniol oxidative pathway led to emergence of the large family of iridoids and seco-iridoids. It was followed by a second elongation, to form MIAs, mainly in the Gentianales lineages of Apocynaceae, Rubiaceae, and Loganiaceae. This pathway is now elucidated up to the formation of the seco-iridoid and strictosidine structures (Irmler et al., 2000; Murata et al., 2008; Geu-Flores et al., 2012; Asada et al., 2013; Miettinen et al., 2014; Salim et al., 2014; Brown et al., 2015). Conversely, in many linalool-producing plants, the linalool oxidation and cyclization lead to the production of a diversity of small cyclic and volatile compounds (Raguso and Pichersky, 1999; Boachon et al., 2015), often stored in the plant as conjugates. It is currently unknown whether cyclic linalool derivatives can be incorporated in more complex structures, equivalent to MIAs.

It is also currently unclear whether cytochromes P450 alone catalyze the formation of cyclic linalool derivatives, such as linalool oxides or lilac aldehydes, or if additional enzymes are needed to synergize oxidation steps or cyclize activated precursors generated by P450s. A human xenobiotic metabolizing P450, CYP2D6, can efficiently convert linalool into cyclic linalool oxides (Meesters et al., 2007). In plants, however, oxidation/cyclisation activity of P450s alone has not yet been confirmed. *A. thaliana* CYP76C1 can form lilac aldehydes from 8-oxolinalool *in vitro* with low efficiency, but its overexpression in *N. benthamiana* was not sufficient to generate detectable amounts of lilac compounds. This could be either due to *N. benthamiana* enzymes competing for the conversion of the intermediates, but may as well signal requirement for additional enzyme(s), which remain to be identified. It is striking that several P450-catalyzed reactions in the terpene-derived metabolism seem to also involve other redundant oxidoreductases that might be required *in vivo* (Paddon et al., 2013; Miettinen et al., 2014; Brown et al., 2015). In addition, none of the P450 enzymes characterized so far has been able to produce pyranoid linalool oxides, in spite of their widespread occurrence in plant kingdom.

A feature common to many CYP76s so far investigated is their low regio- and stereoselectivity. Several members of the CYP76A, B and C family were reported to oxidize a whole subset of monoterpenols, the most striking example being CYP76B6 from *C. roseus* that catalyzes the single or double oxidation of all linear monoterpenols (geraniol, linalool, lavandulol, citronellol) with a similar high efficiency (Höfer et al., 2013). Other examples are CYP76C1 and CYP76C3, both of which metabolize (*R*)- and (*S*)-linalool with no significant enantioselectivity (Höfer et al., 2014; Boachon et al., 2015). In addition, CYP76B1, CYP76C1, CYP76C2, and CYP76C4 were all found to metabolize several phenylurea herbicides. Their herbicide-metabolizing capacity was sufficient to confer an increased herbicide tolerance to plant transformants (Didierjean et al., 2002; Höfer et al., 2014). The low selectivity of CYP76C1 is also illustrated by the diversity of its products, including 8-carboxylinalool, lilac aldehydes or lilac alcohols. This raises the question of promiscuity of these enzymes with regard to other plant metabolites and xenobiotics that have so far not been investigated. In rice, CYP76Ms evolved as multifunctional enzymes dedicated to the biosynthesis of labdane-related diterpenoid antifungal phytoalexins (Wang et al., 2012; Yisheng et al., 2013). In sandalwood (*Santalum album*), CYP76Fs are described as santalene/bergamotene hydroxylases showing relatively broad substrate- and regio-selectivities (Diaz-Chavez et al., 2013). In these cases, as for other described CYP76s (e.g., Guo et al., 2013; Zi and Peters, 2013), only limited functional screening was carried out. More systematic functional investigations and structural studies are thus needed to appraise the specificities and catalytic capacities of the different enzymes.

The monoterpenol oxidative metabolism usually controls the formation of volatile or soluble oxygenated derivatives, in particular in flowers, fruits, and young leaves, which require intensive protection against herbivores and other antagonists or predators. Together with glycosyl transferases, they also control the level of emission of free monoterpenols. There are now several examples where this activity sets the balance between attractiveness for pollinators and repellence of antagonists. In flowers (Knudsen and Tollsten, 1993) and fruits of *Carica papaya* (Schreier and Winterhalter, 1986; Winterhalter et al., 1986; Flath et al., 1990), as well as flowers of *C. breweri* (Raguso and Pichersky, 1999) and *D. mezereum* (Borg-Karlson et al., 1996) linalool and linalool oxides have a demonstrated role in insect attraction. The need for maintaining a very high flexibility of this system (possibly in association with speciation or complex interactions, including tri-trophic plant–insect interactions) could be responsible for the high versatility of this pathway. An alternative hypothesis is that monoterpenol metabolism evolved as a detoxification pathway, since similar pathways are reported in insects and microorganisms (Raguso and Pichersky, 1999). In this case, the broad range of toxic compounds to process would provide another explanation for its high versatility. The ecological role of linalool and its derivatives is already well established, but the influence of iridoids and MIAs on plant adaptation and resistance to antagonists and microorganisms remained largely unexplored, while interest has been focused on pharmaceutical applications of these compounds.

The recent description of the core linalool and geraniol oxidative metabolism in *A. thaliana* and *C. roseus* paves the way to the discovery and engineering of the monoterpenol-derived pathways of plants of agronomic and economic interest, not only for the production of metabolites of interest, but also for a direct plant protection effect. Among the most appealing candidates are fruit, wine and tea aroma, as well as the large diversity of iridoids and alkaloids with documented therapeutic applications. The development of MIAs-producing platforms in microorganisms is currently a very active field of research.

## Author Contributions

Writing first draft and drawing figures: Introduction, monoterpenol derivatives in food and beverages, first insights into geraniol and linalool oxidative metabolism (TI); Geraniol derivatives and iridoids, first insights into geraniol and linalool oxidative metabolism (CP); role of linalool and its derivatives in plant-insect interactions, linalool metabolism (BB); geraniol metabolism (NN), Perspectives and open questions (DW-R). Writing a review and editing (NN and DW-R).

## Conflict of Interest Statement

The authors declare that the research was conducted in the absence of any commercial or financial relationships that could be construed as a potential conflict of interest.
